# A Development of an IoT-Based Connected University System: Progress Report †

**DOI:** 10.3390/s23062875

**Published:** 2023-03-07

**Authors:** Slavomir Matuska, Juraj Machaj, Miroslav Hutar, Peter Brida

**Affiliations:** Faculty of Electrical Engineering and Information Technology, University of Zilina, 010 26 Zilina, Slovakia

**Keywords:** IoT, smart systems, indoor navigation, mobile application

## Abstract

In this paper, a report on the development of an Internet of Things (IoT)-based connected university system is presented. There have been multiple smart solutions developed at the university over recent years. However, the user base of these systems is limited. The IoT-based connected university system allows for integration of multiple subsystems without the need to implement all of them in the same environment, thus enabling end-users to access multiple solutions through a single common interface. The implementation is based on microservice architecture, with the focus mainly on system robustness, scalability, and universality. In the system design, four subsystems are currently implemented, i.e., the subsystem for indoor navigation, the subsystem for parking assistants, the subsystem for smart classrooms or offices, and the subsystem for news aggregation from university life. The principles of all implemented subsystems, as well as the implementation of the system as a web interface and a mobile application, are presented in the paper. Moreover, the implementation of the indoor navigation subsystem that uses signals from Bluetooth beacons is described in detail. The paper also presents results proving the concept of the Bluetooth-based indoor navigation, taking into account different placements of nodes. The tests were performed in a real-world environment to evaluate the feasibility of the navigation module that utilizes deterministic fingerprinting algorithms to estimate the positions of users’ devices.

## 1. Introduction and Motivation

In recent years, Internet of Things (IoT) technology has experienced a global boom. It is currently used in various industrial applications as well as in the daily lives of ordinary people. The fast and efficient development and deployment of IoT applications are enabled by miniaturizing and reducing hardware costs and the existence of various software-oriented services.

In recent years, several independent intelligent systems were developed at the University of Zilina. These systems were either used independently with a limited number of users or were not used at all and have fallen into oblivion. The main motivation for the development of an IoT-based connected university system was to create a versatile and robust system built on microservices so that it will be possible to implement individual solutions as sub-systems within that system.

Taking into account that the individual services may not have anything in common and may be built in completely different architectures and programming languages, the decision was made to build the proposed system using a microservice architecture. In this architecture, the individual microservices can be created in various programming languages, since each service operates in its own container with its own environment variables. Moreover, the advantage of the microservice architecture is that the possible malfunction of a single service does not affect the other services. The proposed system is open for the implementation of novel sub-systems in the future, due to the use of the microservices architecture.

The individual services implemented within the system are intended to assist or facilitate the normal daily routine within the university. As part of the system design, four sub-systems were proposed, while each service will provide benefits for users at the university in a different way. For example, the news aggregation subsystem is designed to cause it to be easier to bring together news from different sources of university life and display it in one place. This subsystem also retrieves current and historical weather information from around the university. Moreover, the subsystem for assisted parking is intended to speed up finding a free parking space, saving time but also saving the environment. On top of that, the smart classroom subsystem is to assist students and staff in following the principles of proper chair seating and, in this way, help to prevent health problems. Furthermore, the indoor navigation subsystem is useful for junior students and helps them navigate the university campus and find the right classroom.

The principles within each subsystem are based on previously published works and thus do not provide novel approaches. However, the main contribution of this paper is the design and implementation of a robust IoT-based connected university system, which connects the different subsystems and causes them to be accessible through a single common interface, i.e., web interface or mobile application. The system, therefore, enables the creation of novel services for a user taking into account data from multiple subsystems.

The remainder of the paper is organized as follows, Section 2 provides a review of the related work on IoT system development, Section 3 describes the proposed system design and architecture, Section 4 presents the subsystems and their implementation, Section 5 describes the system implementation from the web interface and mobile application point of view, Section 6 presents the proof of concept of implemented BLE localization, and Section 7 concludes the paper.

## 2. Related Work

The development of IoT systems and applications is currently of interest to many research groups. IoT systems and applications are widely used in multiple areas. In general, IoT systems and applications collect information from sensors and use the captured data to optimize processes or create insights into the data that can be useful.

A smart air quality monitoring IoT-based infrastructure for industrial environments was presented in [1]. The authors presented a complete air quality monitoring infrastructure based on the IoT paradigm that is fully integrable with current industrial systems. The monitoring infrastructure includes highly precise compact devices to facilitate the real-time monitoring of particulate matter concentrations and polluting gases in the air. The Big Data collected by this system are processed using machine learning techniques to predict whether safety levels might be surpassed.

Anumala et al. [2] described an IoT-based air quality monitoring system to continuously observe the air quality inside a room. The system collects information about the temperature, humidity, dust, and gas level. All the data are stored in the database and also displayed in real time on the LCD in the room. When the measured values exceed threshold values, a warning message is sent to the house owner’s mobile device using the Global System for Mobile communication (GSM) module.

A long-range outdoor air quality monitoring system based on the LoRaWAN was proposed in [3]. The system consists of multiple sensors (NO_2_, SO_2_, CO_2_, CO, PM2.5, temperature, and humidity), an Arduino microcontroller, a LoRa shield, a LoRaWAN gateway, and The Thing Network (TTN) IoT platform. The IoT nodes are powered by a rechargeable battery with a photovoltaic solar panel. The measured data are stored in the cloud and system users can easily access them on the web-based dashboard. The authors validated the collected data against the high-technology Aeroqual air quality monitoring devices and proved that their system can reliably monitor various air quality indicators.

The monitoring system applied to an aeroponic greenhouse based on an IoT architecture was presented in [4]. The system provides information on the status of the climatic variables and the appearance of the crop in addition to managing the irrigation timing and the frequency of the visual inspections using an Android application called Aeroponics Monitor. The authors use the Thingspeak cloud for data analysis and Firebase servers for data storage.

The challenges in agriculture were addressed in [5]. The proposed IoT-based system was developed for monitoring environmental parameters such as soil moisture, temperature, and humidity. The AgriWealth farming system utilizing the IoT sensors and the Android application that helps farmers control and manage the farm was proposed in [6]. The machine learning model is used to predict suitable crops in accordance with varying weather parameters.

Another flexible IoT agriculture system for irrigation control based on Software Services was described in [7]. The presented paper adopts a software-centric perspective to model and design IoT systems in a flexible manner and provided a simple and novel view of the design of IoT systems in agriculture from the software perspective.

Ahmed et al. [8] designed and implemented a flexible IoT-based platform for the remote monitoring of agriculture farms of different scales, enabling continuous data collection from various IoT devices. IoT nodes are based on the LoRaWAN technology. The authors undertook an experimental validation and showed that the platform can be used to obtain valuable analytics of real-time monitoring that enable decisions and actions such as, for example, controlling the irrigation system or generating alarms.

Security is an important aspect of all information systems, especially in IoT. Vashishtha et al. [9] presented security and detection mechanisms in IoT-based cloud computing using a hybrid approach. They combine RSA and RC4 algorithms for the generation of the key to obtain the superior security method. Security vulnerabilities continue to exist, and security incidents continue to increase. Kim and Yoo [10] classified and analyzed the most common security vulnerabilities for IoT devices and identified the essential vulnerabilities of IoT devices that should be considered for security when producing IoT devices.

IoT technology is also used in smart homes. Vishwakarma et al. [11] proposed a smart energy-efficient home automation system that can remotely access and control home equipment. The solution is based on NodeMCU, Adafruit sensors, and the Arduino platform. The design and implementation of a Cloud-IoT-Based Home Energy Management System were presented in [12]. The developed system allows for collecting and storing energy consumption data from appliances and the main load of the home. Two scenarios were designed and an implemented AWS cloud was used to store all the collected data.

Wu et al. [13] proposed a wearable sensor network system for health applications. The proposed network incorporates multiple wearable sensors to monitor the environmental and physiological parameters of individuals. Based on the sensor specification, the implemented sensors communicate using the LoRa network or Bluetooth. The system includes a warning mechanism that is triggered when a harmful environment is detected. Onasanya and Elshakankiri [14] described a Smart integrated IoT healthcare system for cancer care. They proposed the implementation of an IoT-enabled medical system for the enhanced treatment, diagnosis, detection, and monitoring of cancer patients based on cancer care services. They also created business analytics for insights creation, decision-making, data transmission, and reporting.

The authors in [15] described an IoT-connected e-textile wearable for neonatal medical monitoring called NeoWear. The proposed chest belt wearable should monitor the respiration rate and detect apnea events in babies. The NeoWear is a wearable system consisting of a sensor belt, a wearable embedded system, and an edge computing device. The IoT device is connected to the edge computing device and sends the data over the MQ Telemetry Transport (MQTT) protocol. Their findings show an average error of 0.89 BrPM in respiration rate measurement and 97% accuracy in apnea detection on the programmable baby mannequin.

Other important fields, where IoT systems are widely used are smart cities and smart transportation. Zhu et al. [16] presented a vision as well as work on the integration of artificial intelligence and the intelligent transportation system (ITS) to create an enhanced intelligence of IoT-enabled ITS. A design of an IoT-based smart city model on Raspberry Pi was proposed in [17]. Kumar et al. created an urban IoT system utilizing Raspberry Pi to help smart cities solve domestic difficulties. One of the major smart cities issues is energy optimization.

The authors in [18] proposed a model that can be used to optimize energy consumption in smart homes and smart cities alike. They equipped all smart city electric appliances with the sensors, such as street lighting, building and street billboards, smart homes, and smart parking. The suggested model was evaluated using mathematical modeling and the findings indicated that the proposed model may assist in improving energy usage in smart cities. Smart cities also include people who can use their mobile devices to participate in sensing various environmental variables. This is called crowd-sensing.

The authors in [19] proposed privacy-preserving hybrid sensing for smart cities. They explored an integrated paradigm called “hybrid sensing” that harnesses both IoT-sensing and crowd-sensing in a complementary manner. The authors presented their hybrid sensing on the smart system parking, by which users can inquire and find the available parking spaces in outdoor parking lots. IoT solutions are widely implemented as parking sensing systems that deploy robust outdoor vehicle localization and recognition methodology.

In the [20], the authors proposed a new low-cost sensor system allowing for real-time parking occupancy monitoring along with parking payment without the requirement of any user/driver interaction. A two-stage hybrid approach to help drivers find a parking space was presented in [21]. The proposed solution should decrease the time and energy consumed in finding the parking lot. The first stage focuses on car parks with at least one free parking space located near the target address. The most suitable parking space is searched for and presented in the second stage. The authors also proposed a dynamic cloud-based parking lot reservation system.

A smart parking solution based on the integration of NB-IoT radio communication technology and the core IoT platform was proposed by authors in [22]. Their solution for smart parking benefits from the usage of narrow-band Internet of Things (NB-IoT) technology. The authors created a mobile application for the real-time checking of parking lot availability. A parking space can be reserved via a smartphone application, which will help drivers to find and reserve spots, park their vehicles, and pay.

Sobeslav and Horacek [23] presented A Smart Parking System Based on a Mini PC Platform and Mobile Application for Parking Space Detection. The solution relies on an MPU–9250 magnetometer sensor connected to an Arduino mini microprocessor to detect the vehicle in the parking lot. To send data to the Raspberry Pi server, the IQRF, which is a wireless mesh technology in sub-GHz ISM radio bands, was used. It is possible for a user to create parking place reservations via the mobile application and then the application navigates the user to the reserved spot. The problem with this solution is that it needs a lot of IoT nodes to cover larger parking lots. The camera-based solution is more widespread since it is much easier to implement.

The solutions proposed in [24] and ref. [25] describe the real-time parking lot occupancy detection systems based on a visual sensor network. In both papers, a camera-based solution is used and the achieved results proved that this type of system is suitable for parking occupancy detection.

Indoor localization and navigation is still a hot research topic and an active field of development for many researchers. The most accurate indoor positioning systems are based on UWB technology [26]. A comprehensive survey for indoor positioning systems for IoT-based applications was presented in [27]. Their final finding was that the Global Positioning System (GPS) and the indoor positioning system (IPS) can act as complements to offer a comprehensive location-based service for both indoor and outdoor environments.

Schroeer [28] presented a real-time UWB multi-channel indoor positioning system for industrial scenarios. The author uses four transceivers instead of one to increase the system robustness for each base station. The root mean square error has been reduced by 0.1m in comparison with the single-channel indoor positioning system. However, it is necessary to install a based station and equip users or objects with the active tag in order to use the UWB technology for positioning and indoor navigation.

Another option is to use smartphones and their sensors for localization and navigation. Bluetooth localization technology, its principles, applications, and future trends are summarized by the authors in [29]. They reviewed the applications and existing commercial solutions, revealing the possible directions for the industrialization of Bluetooth localization. The Bluetooth Low Energy (BLE)-based indoor localization systems are limited to using only three advertisement channels. The work presented in [30] analyzed the impact of channel diversity on the accuracy of BLE-based indoor localization. The experiments conducted in a 100 m${}^{2}$ office area show that using signal strength measurements in 40 channels improves the average localization accuracy by approximately 50%.

The challenges related to indoor positioning using Wi-Fi signals were summarized in our previous work [31,32]. An improved Wi-Fi location fingerprint positioning algorithm for robot indoor positioning and navigation was described in [33]. In order to eliminate the location fingerprints that degrade the localization accuracy, Ye and Peng integrated an improved adaptive K-value WKNN algorithm at the end of the localization algorithm. The experimental results show that the probability of the improved algorithm’s positioning error within 0.4 m is 49%, which is a 35% improvement over the conventional algorithm. A smartphone-based indoor navigation system was presented in [34]. They present enhancing the particle filter by utilizing a map constraint and k-means clustering and integrating Bluetooth low energy along with pedestrian dead reckoning for positioning. For the overall performance of PFMK, a mean error of <1.5 m in the test environments was achieved.

## 3. IoT-Based Connected University System Design

The IoT-based connected university system is a huge IoT system that allows users to access various services provided at the campus of the University of Zilina. The system includes multiple different subsystems implemented as micro-services while providing the end-users information from the system via a single common user interface (mobile application and web). The proposed subsystems are as follows:

- Subsystem for news aggregation;
- Subsystem for smart assistant parking;
- Subsystem for smart classroom or office;
- Subsystem for indoor navigation.

**Figure 1:**
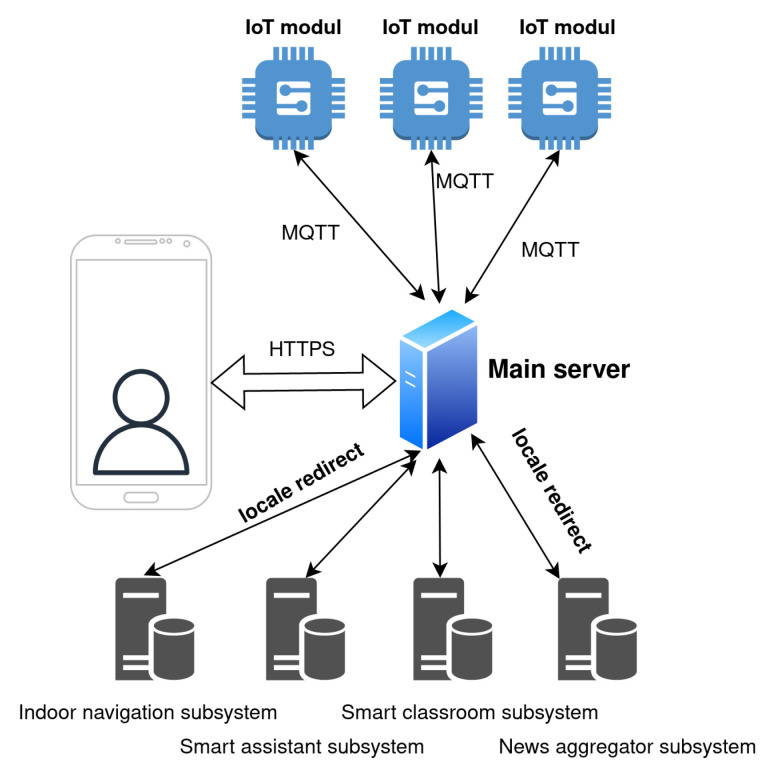
The principle of the system design of the IoT-based connected university.

The design of the system is focused on the robustness, scalability, and universality. The proposed design is open, so it is possible to implement new services in the future. The principle of the system design is illustrated in Figure 1.

**Figure 2:**
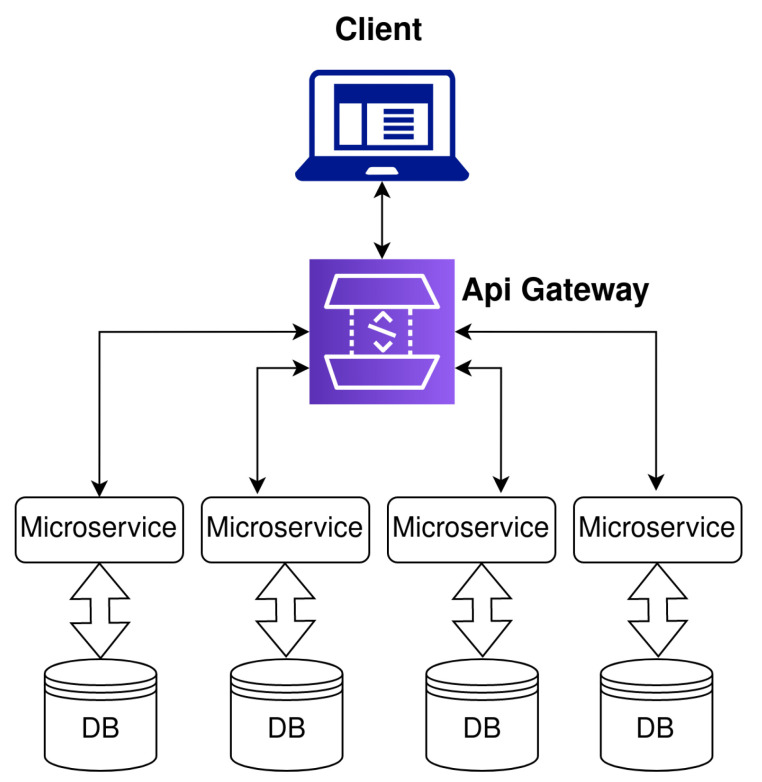
Microservice architecture.

The main unit of the proposed IoT-based connected university system is a server placed on the premises. The users communicate with the server exclusively through HTTPS requests to ensure communication security. Overall, the system provides two levels of users—non-authorized users and authorized users. Some of the services implemented in the system are considered to be free services, which are accessible also to non-authorized users and are available on the home page of the system. However, some subsystems, including indoor navigation and smart classroom subsystems, are only available to authorized users. The system architecture design is based on microservices. Microservices, also known as microservice architecture, is an architectural style that structures an application as a collection of services. The advantage of microservices is that individual services can be independently deployed, are highly maintainable, scalable, and robust, and can be organized around business capabilities. The microservice architecture implemented in the system is shown in Figure 2.

**Figure 3:**
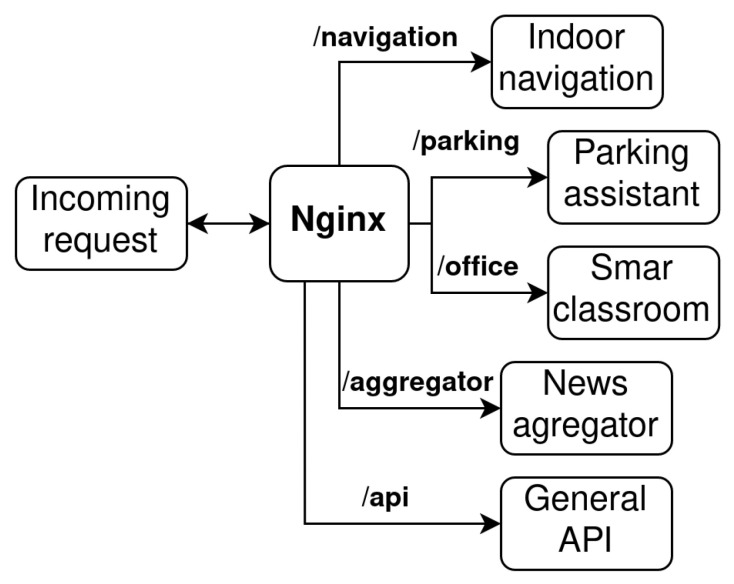
URL-based request routing flow diagram.

The microservice architecture also includes Application Programming Interface (API) gateway and management/orchestration tools. The API gateway represents an entry point for the clients and decouples the clients from the services. In the proposed solution, the Nginx server is used as an API gateway. The Nginx server is responsible for routing the incoming request to the particular service based on the request URL, as can be seen in Figure 3.

The services can also communicate with each other. For this purpose, internal APIs are specified, and the Nginx server is responsible for routing the internal API between the services within the virtual network. Management/orchestration tools are responsible for placing services on nodes, identifying failures, re-balancing services across nodes, and so forth. In the proposed system, Docker and Docker-compose are used for microservices management. The whole system is designed to be ready and compatible for deployment into off-the-shelf technologies, such as Kubernetes. Moreover, it is also possible to use Docker in the swarm mode to boost the scalability of microservices.

## 4. Subsystems Specification

Currently, there are four subsystems designed in the IoT-based connected university system. This section is dedicated to subsystem specification.

### 4.1. News Aggregator

**Figure 4:**
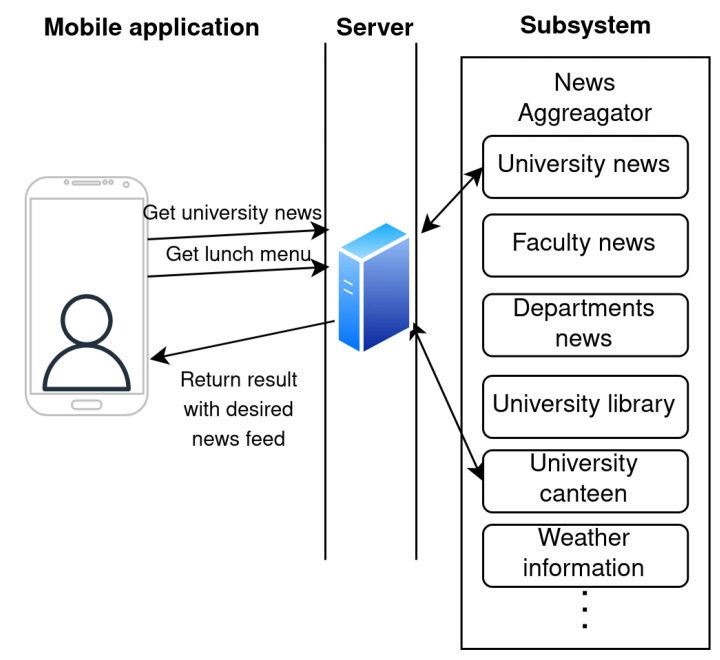
Proposed design of subsystem for news aggregator.

The news aggregator subsystem is a free service, it collects information from multiple sources and provides a single interface to display all of them. The main advantage of this subsystem is that students can find all information in one place. The News aggregator collects the news feeds from multiple university web pages. Many of these are created by a Content Management System (CMS) such as WordPress. The WordPress-based web page provides predefined XML files as an RSS news feed that are easily accessible. However, some of the pages are currently built using a special Joomla CMS plugin and do not provide a news feed. Therefore, it is necessary to parse the page content and search for news. The subsystem also gathers information from the university canteen as well as the university library. In this subsystem, the weather information from local weather stations at the university is also gathered. The source of the weather information is our previously developed system [35] for collecting meteorological data from numerous small weather stations. The users can see weather information from various locations as well as the historical data. The design of the proposed subsystem is shown in Figure 4.

### 4.2. Smart Assistant Parking

**Figure 5:**
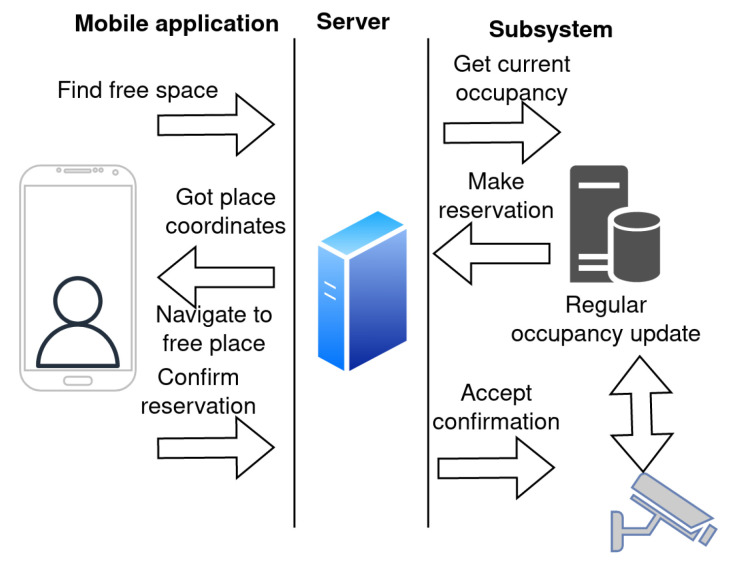
Proposed subsystem for smart assistant parking.

The subsystem for smart assistant parking is one of the free services implemented in the IoT-based connected university system. The goal is to provide information about parking occupancy and navigate the user to the nearest free parking spot. There are two ways how to evaluate parking occupancy, i.e., camera- and sensor-based solutions. In the proposed design, the camera system deployed on the university campus is used to collect information about parking occupancy. The concept of the proposed subsystem is presented in Figure 5. The parking detection is based on a convolutional classifier with a residual architecture [36] that achieved an average accuracy of 98.42 % on the PKLot database.

The application automatically detects that the user has approached the parking area and sends a request to find the free parking place with the current position of the user. Since the university campus covers a wide area, the user is allowed to set their preferred parking place area (e.g., based on the building they are going to). The server redirects the request to the subsystem for the smart parking assistant to receive the current parking lot occupancy. Based on the request and parking occupancy data, the subsystem creates a parking place reservation and sends it in a response message to the client. On the client side, the application receives data and navigates the user to the reserved parking place. The user is allowed to accept or decline the reservation. On the subsystem side, the parking occupancy information is updated on a regular basis.

### 4.3. Smart Classroom or Office

The primary motivation for smart classroom subsystems is to help users who pay attention to their health and proper sitting posture during work or classroom lectures. Moreover, the system is able to estimate the occupancy of offices and lecture rooms based on information from smart chairs. Both students and lecturers spend many hours seated on chairs or stools. Therefore, adopting the correct sitting position is essential for maintaining good posture and a healthy back and spine. There are multiple ways how to monitor the seating posture of individuals. An overview of systems on sitting posture monitoring can be found in [37]. Basically, there are three main approaches used to obtain data about sitting posture:

- Computer image processing;
- Wearable clothing with sensors;
- Measuring the load distribution on some form of substrate.

**Figure 6:**
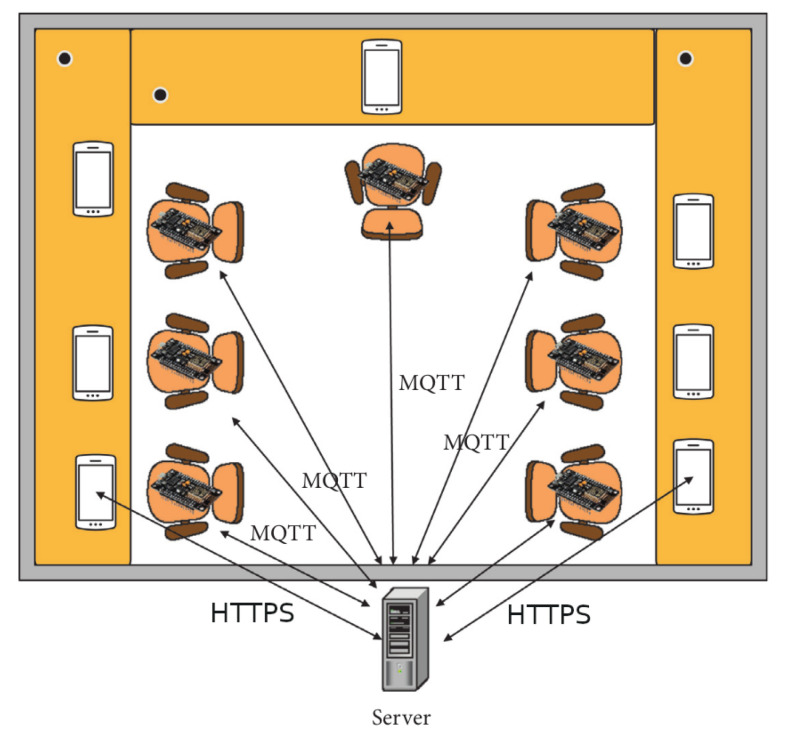
Proposed subsystem for smart classroom.

As part of previous work, a smart system for sitting posture detection was developed. The system is based on force sensors implemented in a chair [38]. The basic concept of the proposed system is illustrated in Figure 6.

In each smart chair, six flexible force sensors FSR402 are implemented. Two sensors are placed in the backrest and four in the bottom seat. A NodeMCU board collects data about changes in the resistance of individual sensors and sends these data to the server using the MQTT protocol. There can be a variable number of chairs in one classroom. On the server side, the system uses the Node-RED application for the evaluation and processing of the data. The user can access information about sitting posture correctness as well as detailed information using the mobile application.

Based on data from the experiments, an algorithm for the evaluation of correct sitting posture was proposed. The algorithm has minimal computation power requirements. The data collected during the testing phase were divided into three groups based on the correctness of the sitting posture. During the evaluation of the results, threshold values were defined for each of the three groups. The typical routine for the individuals in the smart classroom should be as follows:

- The individual chooses a free chair in the classroom;
- Using a mobile application, authorized users scan the QR code of their chair;
- The application provides information about the sitting posture to the user as regular updates.

The design of the smart system for sitting posture detection is easy to implement as a subsystem in the IOT-based connected university system. Therefore, authorized users have access to the data from smart chairs and can keep track of the correctness of their sitting postures.

### 4.4. Indoor Navigation System Design

Bluetooth beacons will be deployed in the buildings of the University of Zilina to collect environmental data. Moreover, the signals from these beacons will be used for the implementation of the navigation subsystem. Since BLE beacons will be implemented in the indoor environment with harsh signal propagation conditions, the implemented localization microservice relies on fingerprint positioning.

The main advantage of fingerprinting localization is that there is no need for distance estimation using a signal propagation model. The fingerprinting localization is based on a comparison of measured data with a radio map database. Since the database was collected in the same environment, fingerprinting localization seems to be immune to multipath signal propagation. However, the drawback of fingerprinting localization is related to the time complexity of radio map measurements.

Since position estimation is implemented in the application created for mobile devices, simple deterministic localization algorithms have to be used. These algorithms are based on an assumption that the received signal strength (RSS) from multiple tags depends on the position of the mobile device. Therefore, the position of the mobile device can be estimated by selecting points with the lowest Euclidean distance between the RSS samples collected during localization and the RSS samples stored in the radio map. More details on algorithms tested during the initial trial will be provided in Section 5.

The primary goal of the indoor navigation subsystem is to provide position estimates based on available Bluetooth signals and deploy an indoor navigation system to help the students with orientation inside the university campus. This is a crucial service especially for new students, as they often find it difficult to navigate through the tangle of corridors and arrive in time for lectures or laboratory exercises.

Therefore, the use of the mobile application with data from this subsystem can help students with finding the right route to the desired lecture room. The subsystem is implemented as an independent service in the mobile application. The application requires permission to use a Bluetooth device on the smartphone in order to be able to obtain the position estimates.

**Figure 7:**
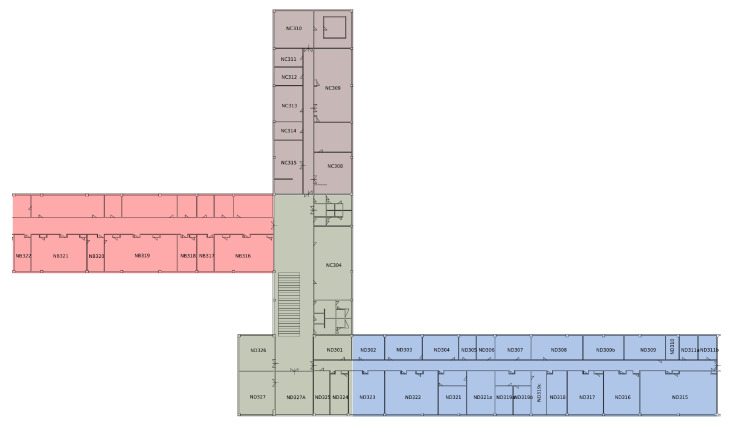
The example of floor divided into the sub-regions.

To implement indoor navigation in a complex building on a university campus, we decided to split buildings into sub-regions, in order to reduce computational complexity related to position estimation. The example of the one-floor splitting is shown in Figure 7. The particular sub-regions are highlighted in different colors.

**Figure 8:**
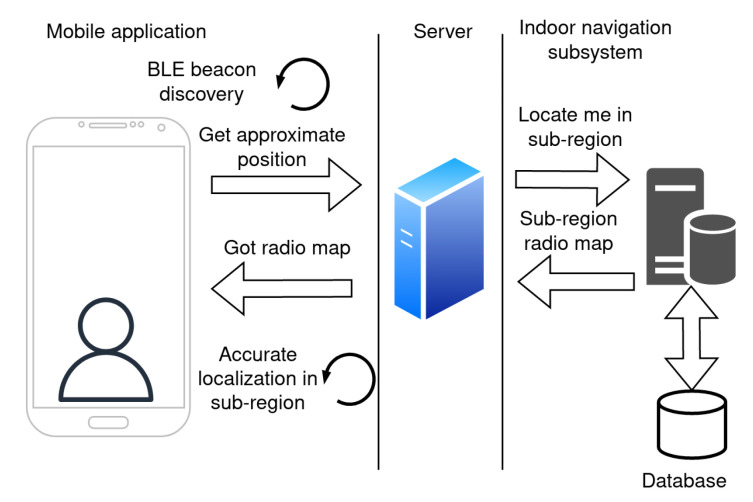
Subsystem for indoor navigation initial localization.

The positioning is performed within these small sub-regions using the fingerprinting method. When the application service on the mobile device for localization is activated, the application estimates an approximate position on the campus. This process is explained in Figure 8.

As a first step, the application will scan for signals from the BLE beacons. The data about BLE beacons in the area are sent in the request to the server for the approximate position in the term of sub-region. The server estimates the sub-region based on the signals from the surrounding beacons. The server response contains information about the sub-region and its radio map. The application then uses a positioning algorithm for accurate localization within the sub-region.

Once the position of the mobile device is estimated, the user can search for the destination room by choosing a room label from the list. The actual list of rooms is provided by the server. Once the room is selected, the application sends the request to find the route to the server. The server response contains data about the navigation route. The mobile application receives the response and initializes the navigation within a single sub-region. Once the user moves, the application periodically updates the position.

**Figure 9:**
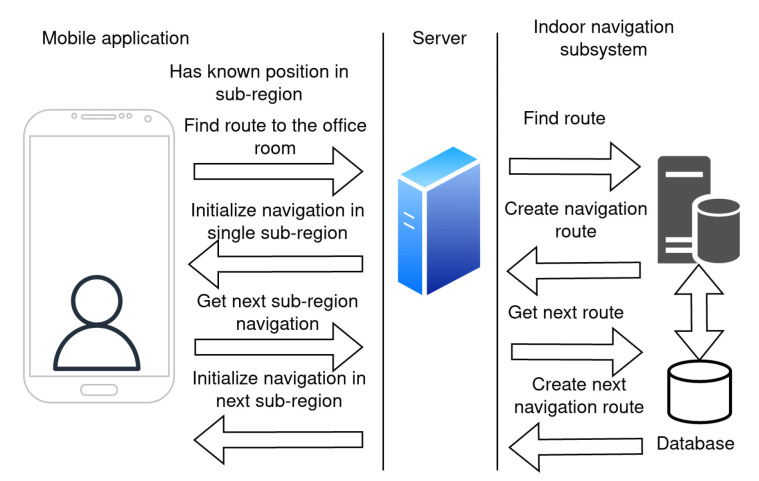
The process of indoor navigation in the mobile application.

When the user approaches a neighboring sub-region, the application sends a request to the server to download data from the next sub-region for localization and navigation. This is repeated until the user reaches the destination sub-region, where they will be pointed to the desired office. The navigation process is depicted in Figure 9.

## 5. System Implementation

In this section, the implementation of the microservices of the IoT-based connected university system will be described in detail. To develop the proposed system, the Docker platform was used. Docker is a platform that allows developers to easily create, deploy, and run applications in containers. Containers represent lightweight, portable, and self-sufficient executable packages that include everything needed to run an application, including the code, runtime, system tools, libraries, and settings.

In the proposed architecture, multiple containers are used. The Docker Compose tool was utilized to handle the containers, networks, and services required for the proposed solution. This tool enables the specification and execution of multi-container Docker applications. It offers a convenient way to manage the entire process of a multi-container application, from development to production. Therefore, it is easy to run the same application in different environments. It is an excellent resource for development, testing, and deployment. This tool is using the docker-compose.yml file for the system setup. Used docker-compose.yml file contains the following services:

- Nginx—entry point to the system;
- MongoApi—serves as storage for the API service;
- API—backend for setting and authentication usage;
- Web—holds React web application;
- Aggregator—implementing logic for news aggregator subsystem;
- MongoAggregator—serves as storage for the aggregator service;
- Localization—implementing logic for indoor navigation subsystem;
- MongoLocalization—serves as storage for the localization service;
- Mongo-express—for development purposes only.

The system starts with the single command “docker-compose up” or “docker-compose up-d” to run the system in the background. During the first run, a single administration account is created with a default username and password, which should be changed after the initial login. The administrator has to set up other services to ensure they work properly. After the initialization process, the IoT-based connected university system is ready to be deployed.

### 5.1. Web-Based User Interface

**Figure 10:**
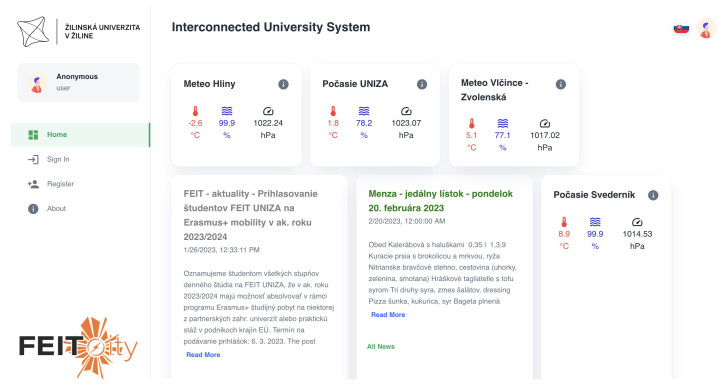
The IoT system for connected university home page for the non−authorized user.

All communication from the Internet is routed via port 3050. The communication then passes through the Nginx server. In the Nginx server, URL base routing is implemented, see Figure 3. The User Interface (UI) was implemented using React—a JavaScript library with the utilization of Material UI (MUI) components. The home page of the IoT-based connected university system for the non-authorized user is shown in Figure 10.

**Figure 11:**
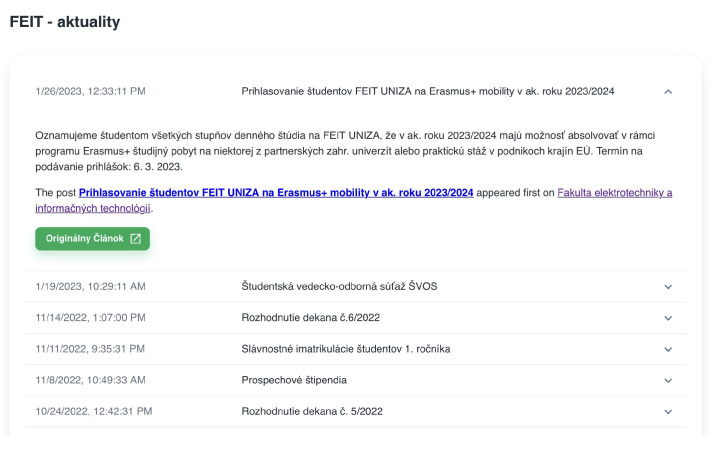
Detailed information for item−type news from aggregator service.

**Figure 12:**
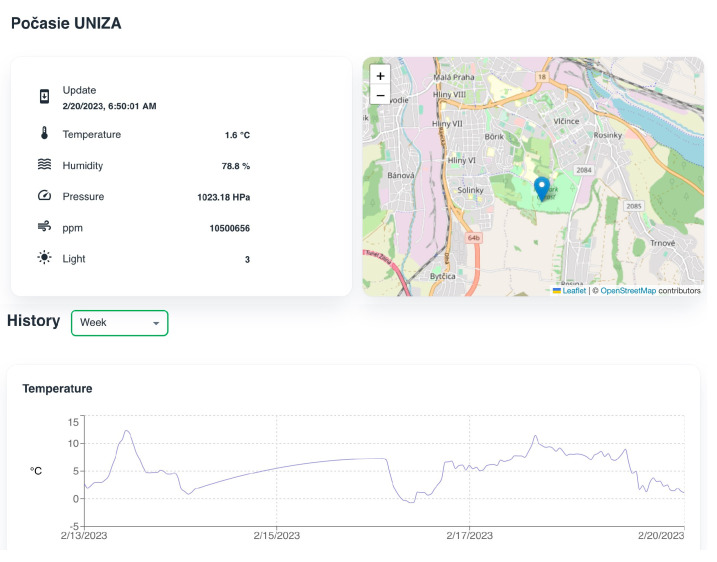
Detailed view of meteorological data.

The non-authorized user can only access free services, such as feeds from news aggregators. The user can manage the displayed services. For the non-authorized user, all settings are stored in the browser’s local storage. Detailed information for each item can be displayed by clicking on it. The detailed news feed is shown in Figure 11 and a detailed view of the meteorological data is shown in Figure 12.

**Figure 13:**
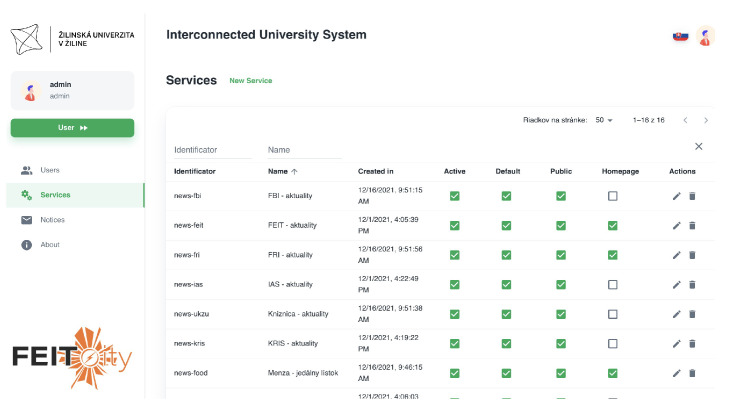
System administration section.

The system also provides an administration section, where the system administrator can manage all the services, users, and notices. The view on the administration sections with settings of the individual services is shown in Figure 13.

As can be seen from the figure, the administrator is able to set different flags for each service, such as an active flag, default flag, homepage flag, or public flag. Each service has a custom identification assigned, in order to identify the service in the system architecture. The name of the service is displayed as the service name on the user’s home screen. Some of the services may not be visible in a web application. An example of such a service is indoor navigation since the native mobile application is required to collect the data required for the proper function of the service.

### 5.2. Mobile Application

A mobile Connected UNIZA application was developed in order to improve the user experience and provide services with added value. The application was developed using the React Native framework. React Native is a JavaScript framework for building mobile applications using React. It allows developers to build mobile apps for iOS and Android platforms using a single codebase, leveraging the power of React and its component-based architecture. React Native uses native components rather than web components as building blocks and allows developers to access the device’s native APIs, such as the camera, GPS, and more. The framework is open-source and actively maintained by Facebook and the community.

**Figure 14:**
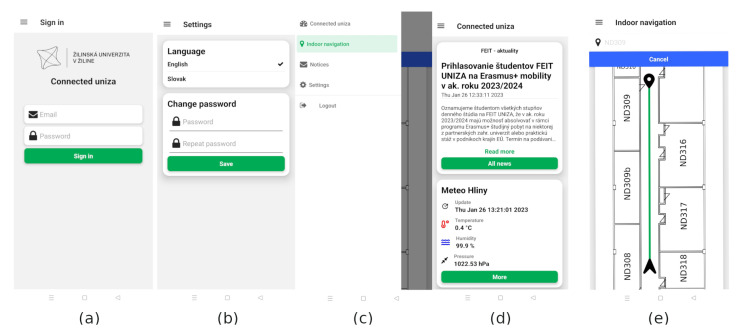
The mobile application of the connected university system, (**a**) Login screen. (**b**) Settings screen for authorized users. (**c**) Navigation drawer. (**d**) Connected university home screen showing news feeds from the aggregator subsystem. (**e**) Indoor navigation screen.

The screens of the developed application are shown in Figure 14. The application is available only for authenticated users. The first screen after the application startup is a login screen, as can be seen in Figure 14a. The user must fill in the credentials in order to login into the application. The users are also able to create an account using the register screen. The home screen for the authorized user is shown in Figure 14d. By default, all services with the “homepage” flag are shown for the user who is logged in for the first time. Then, the user can alter which services will be displayed in the feeds. The authorized users are allowed to change and save the layout of the feed for the next login. The user interface of the application is implemented in two languages, Slovak and English. Users can change the language and password in the settings screen, see Figure 14b. The navigation between the screens is implemented via a drawer navigator, shown in Figure 14c. An example of an indoor navigation screen is shown in Figure 14e. The user can choose the destination room number and the application will navigate the user toward the desired destination.

## 6. Proof of Concept for Indoor Localization

In order to evaluate the feasibility of BLE-based localization for indoor navigation purposes, the preliminary tests were performed at a typical corridor in one of the buildings at the University of Zilina. The corridor has dimensions of 2 × 69 m. The reference points used for radio map measurements were placed in a grid with a spacing of 1 m, resulting in a radio map with measurements recorded at 134 points. The corridor was covered by five BLE beacons. The Holyiot nRF51822 Bluetooth 4.0 beacon BLE module was used. For testing purposes, the position was estimated at 21 positions. The positions of reference as well as the testing points were estimated using a laser distance measurement tool.

**Figure 15:**
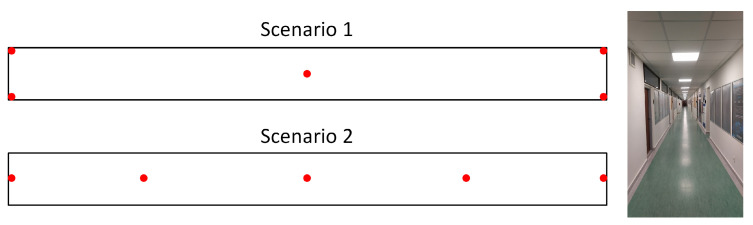
Placement of BLE beacons in testing scenarios.

The tests were performed in two scenarios, and the positions of the BLE beacons in these scenarios are presented by red dots in Figure 15, together with a view of the corridor. In both scenarios, the BLE beacons were attached to the ceiling, so the signal should not be obstructed by other users present in the area.

For testing purposes, the positioning was performed using NN, KNN, and WKNN algorithms. In all these algorithms, the position estimate $\bar{x}$ is provided by:

(1) $$\bar{x}=\frac{\sum_{i=1}^{M}\omega_{i}p_{i}}{\sum_{j=1}^{M}\omega_{j}},$$

where $p_{i}$ is the position of the *i*-th reference point in the radio map, while $\omega_{i}$ and $\omega_{j}$ are weights assigned to the *i*-th and *j*-th reference points and *M* is the number of reference points in the radio map. The weights are calculated as the inverse value of the Euclidean distance between RSS measurements from the mobile device and radio map.

The Nearest Neighbour (NN) algorithm takes into account only the reference point with the highest weight, i.e., the smallest Euclidean distance between RSS samples. The *k*-Nearest Neighbour (KNN) algorithm uses the *k*-highest weights and sets them to 1, while the other weights are set to 0. Therefore, KNN estimates the position as the center of gravity of the selected reference points. Similarly, the Weighted *k*-Nearest Neighbor (WKNN) considers the *k* reference points with the highest weights; however, the weights are considered in the final estimate, i.e., the estimate is calculated as the weighted average of the selected reference points.

**Figure 16:**
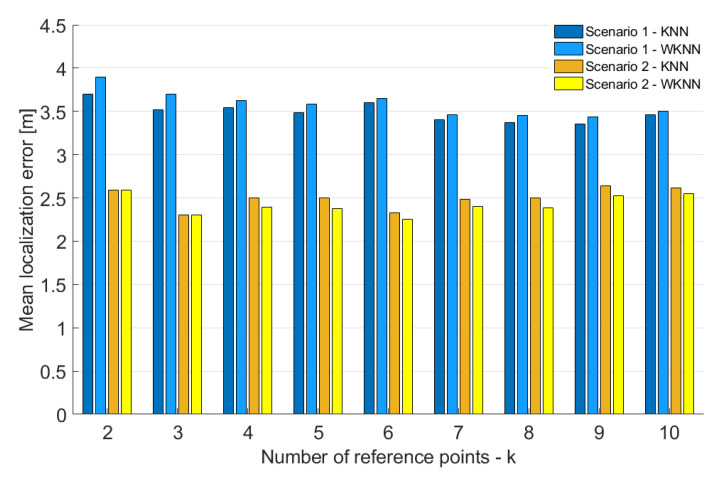
Localization Impact of a number of the refernece points on accuracy of KNN and WKNN algorithms.

In the first step, the impact of a number of the reference points *k* in both KNN and WKNN algorithms was evaluated for both scenarios. The achieved results are shown in Figure 16.

From the figure, it is clear that, for scenario 2, the best accuracy was achieved when the number of reference points used for position estimation *k* = 3. On the other hand, in scenario 1, the best results were achieved with *k* = 9. However, it is important to note that the impact of a number of reference points used for position estimation in both scenarios is relatively small. Moreover, the results achieved in scenario 1 were in all cases significantly worse than in scenario 2. The difference in the mean error between the scenarios was between 0.7 and 1.4 m. That means the placement of BLE nodes in scenario 2 helped to improve the accuracy by 21–37% compared to scenario 1. Based on these results, the number of reference points considered in the KNN and WKNN algorithms was set as *k* = 3 for further investigation. Moreover, it was already proven that the number of reference points *k* = 3 provides reasonably good results using data from Wi-Fi networks [39].

**Figure 17:**
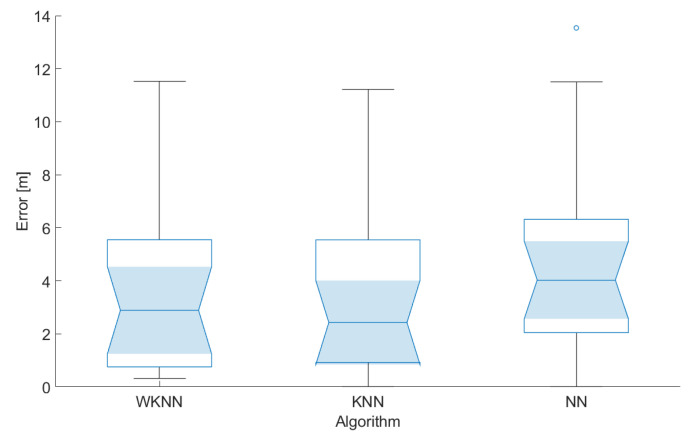
Localization errors achieved in scenario 1.

**Figure 18:**
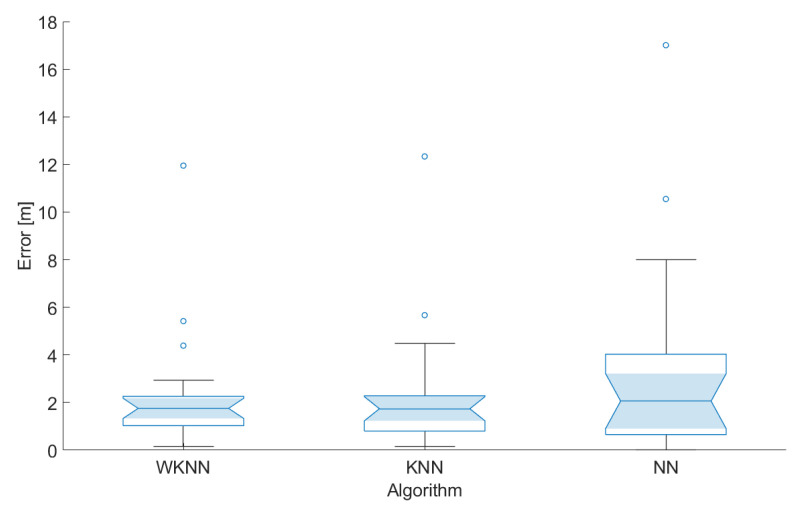
Localization errors achieved in scenario 2.

The results achieved during the testing of the localization concept in scenarios 1 and 2 are shown in Figure 17 and Figure 18, respectively. In the figures, the median is shown as a line inside the box, the 5% confidence interval is represented by the shaded area, the lower and upper quartiles define the edge of the box, the minimum and maximum values that are not outliers are presented by whiskers, and circles represent the outliers in the data.

From the achieved results, it can be seen that the localization errors achieved in the second scenario were significantly smaller, although there were some outliers. This might be caused by the fact that the BLE nodes in the second scenario are distributed more evenly across the area, therefore providing stronger signals from multiple sources.

It can also be concluded that the NN algorithm achieved the lowest performance, i.e., the highest localization error, in both cases. This is due to the fact that part of the test points, used for the evaluation of the positioning algorithms, were placed at different positions as reference points in the radio map.

The best results were achieved using the KNN localization algorithm; in the second test scenario, therefore, this configuration will be considered in the further development and evaluation of the system. Based on these preliminary results, it can be concluded that BLE-based positioning has the potential to provide the accuracy that should be sufficient for navigation in corridors along the university campus.

## 7. Conclusions and Future Work

The paper presented the design and implementation of a functional IoT-based connected university system—a robust and massive IoT system for integrating various services as subsystems. In the pilot design, we proposed four subsystems, i.e., the subsystem for indoor navigation, the subsystem for parking assistance, the subsystem for smart classrooms or offices, and the subsystem for news aggregation from the university. In the initial system implementation, the basic system functionality, the subsystem for news aggregation, and the subsystem for indoor navigation were developed. The subsystems are implemented using the microservice architecture and, thus, are reliable, scalable, highly maintainable, and independently deployable. The system design is open to implementing new services in the future.

In this paper, the implementation of the system was described in detail. Moreover, the performance of the BLE localization microsystem was tested to prove the proposed system design. Based on the achieved results, it can be concluded that the positioning system can achieve reasonable localization accuracy with the correct placement of the BLE beacons and the system can provide position estimates with an accuracy of 2 m. In this paper, different placements of BLE beacons and their impacts on the accuracy of the positioning algorithms were investigated. From the results, it can be concluded that the positions of the BLE beacons have a significant impact on the achieved accuracy. The localization error was decreased by 31% by a change in the BLE node placement. Therefore, it can be concluded that, with the correct placement of BLE beacons, the implemented positioning solution is suitable for navigation in university buildings using low-cost beacons and off-the-shelf smartphones.

In the future, the system will be tested by a limited number of users to evaluate its functionality in realistic daily use. The sensors with BLE communication modules will be implemented in university buildings to gather environmental data and support positioning services in all corridors. The system will also be extended with novel services based on the analysis of data from connected sensors.

## Data Availability

The data presented in this study are available on request from the corresponding author.

## Abbreviations

The following abbreviations are used in this manuscript:

IoT	Internet of Things.
AWS	Amazon Web Services.
MQTT	MQ Telemetry Transport.
GPS	Global Positioning System.
UWB	Ultra-Wide Band.
BLE	Bluetooth Low Energy.
HTTPS	Hypertext Transfer Protocol Secure.
API	Application Programming Interface.
URL	Uniform Resource Identifier.
CMS	Content Management System.
RSS	Received Signal Strength.
UI	User Interface.
MUI	Material User Interface.
NN	Nearest Neighbors.
KNN	K-Nearest Neighbors.
WKNN	Weighted k-Nearest Neighbor.
DOAJ	Directory of Open Access Journals.
TLA	Three Letter Acronym.
LD	Linear Dichroism.
